# Subventricular Zone Radiation Dose and Outcome for Glioblastoma Treated Between 2006 and 2012

**DOI:** 10.7759/cureus.3618

**Published:** 2018-11-21

**Authors:** Sonja C Murchison, Bradley Wiksyk, Stacey Gossman, Brigit Jensen, Dorothy Sayers, Mary Lesperance, Pauline T Truong, Abraham Alexander

**Affiliations:** 1 Radiation Oncology, British Columbia Cancer Agency – Vancouver Island Centre, Victoria, CAN; 2 Internal Medicine, University of British Columbia, Vancouver, CAN; 3 Oncology, University of Victoria, Victoria, CAN

**Keywords:** glioblastoma, subventricular zone, radiotherapy, stem cells

## Abstract

Objective

Stem cells residing in the subventricular zone (SVZ) may be related to recurrence, potentially affecting outcome in glioblastoma (GBM). This study investigated the relationship of SVZ radiation dose and survival in a large cohort treated with surgery and chemoradiotherapy (CRT).

Methods

Patients with GBM treated between 2006 and 2012 (n = 370) were identified. SVZs were contoured from planning computed tomography (CT) with magnetic resonance imaging (MRI) registration where available. Dose was extracted from dose volume histograms. Kaplan-Meier (KM) progression-free survival (PFS) and overall survival (OS) estimates were compared with log-rank tests for SVZ doses. Multivariate analysis (MVA) identified clinical and treatment-related factors significantly associated with outcome.

Results

Median follow-up was 16.4 months, 48.1% underwent gross total resection (GTR), 37.5% subtotal resection, and 14.4% biopsy without resection. Median PFS was 8.9 months (95% CI: 8.3–9.8 months), and OS was 16.5 months (95% CI: 15.2–17.6 months). PFS was significantly lower for older age (>50 years, P = 0.045), poor Karnofsky performance status (KPS, P = 0.049), multifocality (P < 0.001), and incomplete adjuvant chemotherapy (P < 0.001). Worse OS was associated with poor KPS (P = 0.001), biopsy only (P = 0.003), multifocality (P = 0.009), and failure to complete adjuvant chemotherapy (P < 0.001). SVZ dose was not associated with outcome for any of the dose levels assessed. On MVA, multifocality was associated with worse PFS (P < 0.01). Poor performance status and biopsy only were associated with worse OS (both P < 0.01).

Conclusion

In this analysis of a large cohort of GBM treated with surgery and CRT, increased SVZ dose was not associated with improved survival.

## Introduction

Standard treatment for glioblastoma (GBM) in patients younger than 65–70 years involves maximal surgical resection plus radical long-course chemoradiotherapy, followed by adjuvant chemotherapy with temozolomide [1]. Despite aggressive multimodality therapy, prognosis remains poor, with a median overall survival of 14.6 months, and two-year overall survival of 27%.

Tumor-like stem cells (TLSC) exhibit properties that help maintain and promote tumor growth [2,3]. TLSC have been isolated and extensively studied in GBM [2-5]. Research suggests they may contribute to negative outcomes associated with this disease. In both cultured cells and mice models, neural stem cells (NSC) and TLSC both express CD133, which is correlated with greater radioresistance, repopulation, and DNA damage checkpoint response [6]. Disrupted K-ras signaling, a biologic regulator of NSC, has been shown to induce gliomatosis [7]. Mutations in epidermal growth factor receptor (EGFR) have also been encountered in glioma, and in NSC, confer a proliferative advantage and enhanced tumor cell survival [8]. One well-characterized reservoir of NSC in humans is the subventricular zone (SVZ) [9-12]. In some studies, tumor proximity or involvement of the SVZ has been related to poorer prognosis [13-15].

In recent years, there has been speculation as to whether irradiation of NSCs in the SVZ may improve outcome. Unfortunately, there is limited data on the impact of dose to the  SVZ, which consists of mostly small, retrospective studies with conflicting results [16-26]. Some of these studies suggest that higher dose to the SVZ is associated with better outcome, raising the possibility that targeted inclusion of this area into the treatment volume may improve survival [20, 22-26]. The largest of these studies assessed 173 subjects, and found that a higher ipsilateral SVZ dose correlated with better progression-free survival (PFS) and overall survival (OS) [25]. The next largest study involved 116 subjects, but found a benefit only within patients who underwent gross total resection (GTR) [26]. The SVZ is anatomically close to the hippocampal formation, and since irradiation of this region is potentially toxic [27,28], a clearer understanding of the potential benefit of targeting the SVZ is warranted. This study investigates whether SVZ dose is correlated with survival outcomes in a large cohort of GBM patients treated with radical long-course CRT and concomitant temozolomide.

## Materials and methods

Patients

The patients in this study received treatment at an institution that provides all radiotherapy services provincially. This study was approved by the institutional research ethics board. Between 2006 and 2012, all patients above age 18 with pathologically proven GBM treated at the institution with long-course CRT, who completed the full course of radiotherapy and at least 50% of the concurrent chemotherapy, were retrospectively reviewed (n = 370). This study period was chosen to allow sufficient follow-up time (minimum one year) to observe the primary endpoint of PFS. All had initial surgery with GTR, subtotal resection (STR), or biopsy, which was followed by adjuvant radiation (60 Gy in 30 fractions, intensity modulated radiotherapy (IMRT) or 3D-CRT) and at least 50% of the prescribed concomitant temozolomide. Patients were excluded if they did not complete CRT, if full dosimetry data was unavailable, or if the intended final dose was less than 59.4 Gy.

Data collection

Clinical data was extracted from an integrated electronic charting system. SVZs were retrospectively contoured on patients’ planning CT scans, in accordance with operational definitions outlined in previous protocols [23,24] as 5 mm along the lateral wall of the lateral ventricles for all treatment plans, with the use of co-registered magnetic resonance imaging (MRI) where available. This was done by two radiation therapists and one radiation oncology resident with training in SVZ contouring, and reviewed by a radiation oncologist specializing in central nervous system tumors. Dosimetry was analyzed with the analytical anisotropic algorithm on the Eclipse Planning System (Varian, version 11). Dose-volume histogram (DVH) data was collected for the ipsilateral, contralateral, and bilateral SVZ.

Statistical analysis

Statistical analysis was completed with R Version 3.2.5 (R Foundation, Vienna). The primary endpoints were PFS, defined as the time from histologic confirmation of GBM until radiologic evidence of disease progression and a change in patient management, and OS, until death. Kaplan-Meier (KM) curves of PFS and OS were constructed, comparing patients by dose to ipsilateral, contralateral, and bilateral SVZ. Known prognostic factors were also evaluated. KM curves were compared using the log-rank statistic, two-tailed, with P ≤ 0.05 denoting significance. For PFS, patients were censored at the time of last imaging showing stability if they had not progressed at the time of analysis. For OS, patients were censored at their last follow-up appointment or clinical investigation.

For univariate analysis of prognostic factors, patients were grouped by age (<50 vs. >50), tumor location (frontal, temporal, parietal, occipital, cerebellar, butterfly, other), Karnofsky performance status (KPS, >70, <70), multifocality, resection (GTR, STR, biopsy), completion of concurrent chemotherapy, completion of adjuvant chemotherapy (>26, <26 weeks), type of progression (local, distant, both), and time from diagnosis to CRT (<median, >median). The selected cut off for age has been used in similar studies of SVZ irradiation [23, 25]. SVZ dosimetric data was evaluated for groups defined by cut-off points that included median dose, 40 Gy, and 59.4 Gy. The 40 Gy threshold was selected because SVZ doses in the range of 30–43 Gy have been suggested to be of prognostic significance [24, 26]. The 59.4 Gy threshold was evaluated due to the postulated radio-resistance of NSCs [25].

Multivariate Cox proportional hazards (CPH) analysis was performed to identify independent predictors of PFS and OS. Variables included SVZ dose, age at diagnosis, biopsy only, Karnofsky performance status, multifocality, and adjuvant chemotherapy. All covariates, which were known prognostic factors for GBM patient outcome, were included regardless of their significance in the univariate analysis.

## Results

Median follow-up time was 16.4 months for the cohort. Three-hundred sixty of the initial 370 patients were included in the analysis. Nine with incomplete dosimetric data, and one with an unconventional radiation prescription (neither 59.4 Gy nor 60 Gy) were excluded. Baseline characteristics of the group are shown in Table 1. Dosimetric data including quartile doses are listed in Table 2. Median PFS for all patients was 8.9 months (95% CI: 8.3–9.8 months), and OS was 16.5 months (95% CI: 15.2–17.6 months).

**Table 1:** Patient demographics, disease, and treatment characteristics. MRI: Magnetic resonance imaging; CRT: Chemoradiotherapy; IMRT: Intensity modulated radiotherapy; SD: Standard deviation.

Characteristic	N = 360	
Sex		
Female	118	(32.8%)
Male	242	(67.2%)
Age (y)		
< 50	83	(23.1%)
> 50	277	(76.9%)
Tumor location		
Frontal	113	(31.4%)
Temporal	119	(33.1%)
Parietal	84	(23.3%)
Occipital	22	(6.1%)
Butterfly	16	(4.4%)
Other	6	(1.7%)
Karnofsky performance status		
> 70	280	(77.8%)
< 70	80	(22.2%)
Multifocal		
No	301	(83.6%)
Yes	59	(16.4%)
MRI size		
< Median	180	(50%)
> Median	180	(50%)
Resection		
Gross total resection, GTR	173	(48.1%)
Subtotal resection, STR	135	(37.5%)
Biopsy	52	(14.4%)
Adjuvant chemotherapy completed		
>26 Weeks	176	(48.9%)
< 26 Weeks	184	(51.1%)
Concurrent chemotherapy completed		
Yes	339	(94.2%)
No	21	(5.8%)
Progression type		
Local	257	(71.4%)
Distant	11	(3.1%)
Both	42	(11.7%)
No evidence of progression	50	(13.9%)
RT technique		
3D-CRT	264	(73.3%)
IMRT	95	(26.4%)
Other	1	(0.3%)
Total dose (cGy)		
5940	37	(10.3%)
6000	323	(89.7%)
Time from Dx to RT start (d)		
Mean	41.02	
SD	14.27	
MRI largest dimension (cm)		
Mean	4.43	
SD	1.49	

**Table 2:** Radiation dosimetric data summary. SVZ: Subventricular zone

	Ipsilateral	Contralateral	Bilateral
Mean SVZ dose (cGy)			
Mean	4778	2892	3790
Median	4900	2806	3780
1st Quartile	4151	1952	3146
3rd Quartile	5606	3751	4416
Minimum SVZ dose (cGy)			
Mean	1835	908.9	845.1
Median	1462	524.2	448
1st Quartile	345	179.6	173.1
3rd Quartile	2852	1426	1341
Maximum SVZ dose (cGy)			
Mean	3158	4846	6158
Median	6136	5222	6145
1st Quartile	6070	3851	6073
3rd Quartile	6250	5974	6254

**Figure 1:**
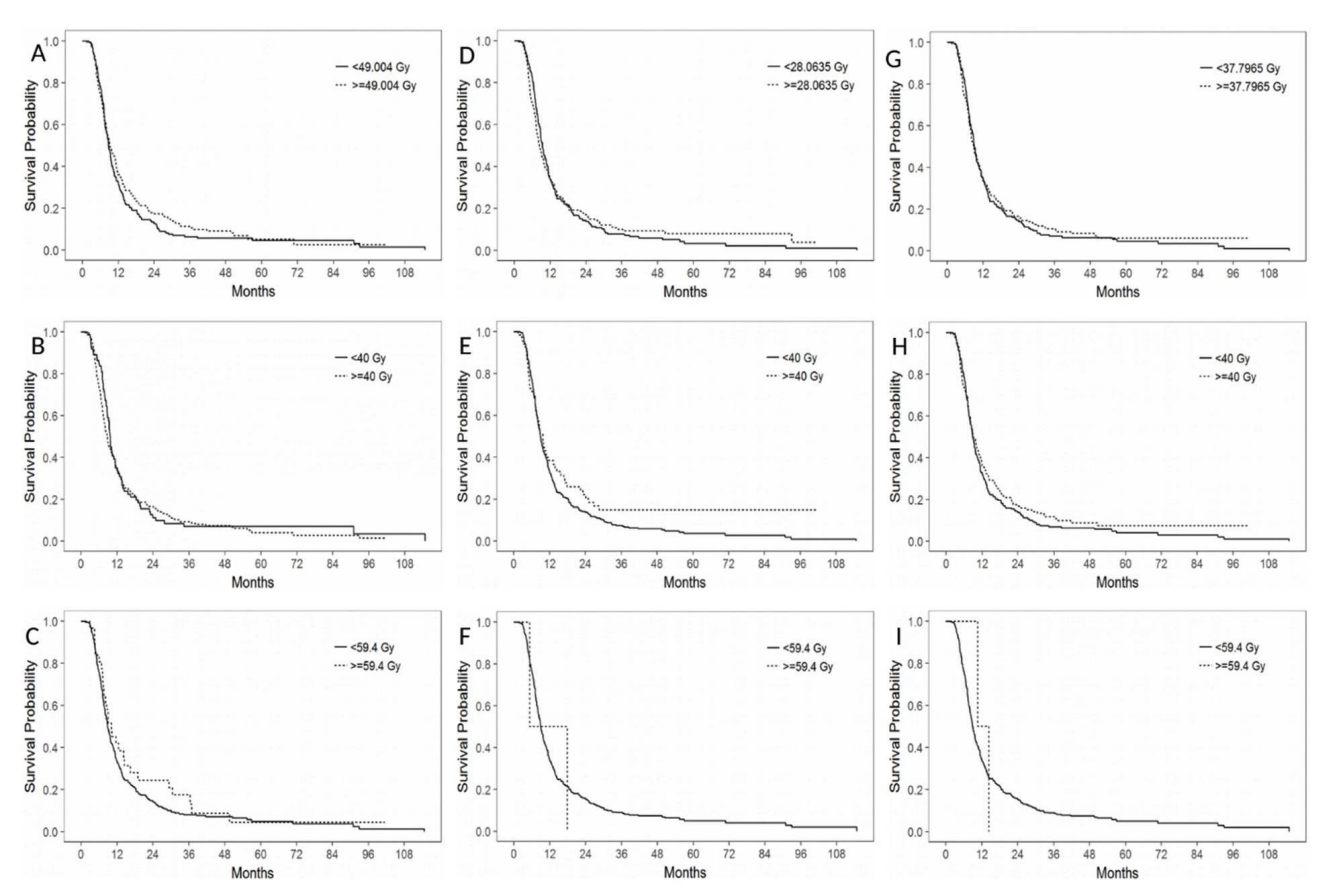
Progression-free survival according to dose to the ipsilateral (A: median dose, B: 40 Gy, C: 59.4 Gy), contralateral (D: median dose, E: 40 Gy, F: 59.4 Gy), and bilateral (G: median dose, H: 40 Gy, I: 59.4 Gy) subventricular zones.

On univariate analysis of all patients (Table 3), worse PFS was significantly associated with older age (P = 0.045), lower KPS (P = 0.049), multifocality (P < 0.001), and failure to complete adjuvant chemotherapy (P < 0.001). Shorter OS was associated with lower KPS (P = 0.001), biopsy rather than resection (P = 0.003), multifocality (P = 0.009), and failure to complete adjuvant chemotherapy (P < 0.001). Dose was not significantly associated with either PFS (Figure 1) or OS, for any of the cut-off points assessed, for ipsilateral, contralateral, and bilateral SVZ, even when analysis was limited to patients who had GTR (Table 3).

**Table 3:** Univariate analysis of prognostic factors associated with PFS and OS. PFS: Progression-free survival; OS: Overall survival; SVZ: Subventricular zone; GTR: Gross total resection; STR: Subtotal resection.

		PFS		OS	
Prognostic Factors		Median (months)	P-value	Median (months)	P-value
Age	< 50 years	10.4	0.045	17.8	0.234
	> 50 years	8.7		16.0	
Tumor location	Frontal	8.8	0.083	17.3	0.057
	Temporal	9.9		17.0	
	Parietal	8.0		14.5	
	Occipital	9.6		17.6	
	Butterfly	11.7		20.3	
	Other	14.4		13.6	
Karnofsky score	> 70	9.7	0.049	17.1	0.001
	< 70	7.2		11.3	
Multifocality	No	9.5	<0.001	16.8	0.009
	Yes	7.1		13.7	
Resection	GTR	9.5	0.245	17.2	0.003
	STR	8.8		17.0	
	Biopsy	6.9		10.7	
Adjuvant chemotherapy	>26 weeks	13.6	<0.001	23.7	<0.001
	< 26 weeks	6.2		10.6	
Concurrent chemotherapy	Completed	9.0	0.535	16.5	0.615
	Incomplete	8.3		17.8	
Progression type	Local	8.3	0.321	17.0	0.589
	Distant	9.9		15.0	
	Both	9.8		12.8	
Time from diagnosis to RT (median)	< 39 days	9.0	0.864	16.5	0.664
	> 39 days	8.8		16.6	
SVZ dosimetric data					
Ipsilateral dose	< 49.0 Gy (median)	8.7	0.251	16.9	0.618
	> 49.0 Gy (median)	9		15.7	
	< 40 Gy	9.6	0.594	17.4	0.259
	> 40 Gy	8.7		15.6	
	< 59.4 Gy	8.8	0.240	16.5	0.194
	> 59.4 Gy	10.1		18.1	
Contralateral dose	< 28.1 Gy (median)	9.4	0.981	17	0.569
	> 28.1 Gy (median)	8.4		15.4	
	< 40 Gy	8.9	0.129	16.6	0.639
	> 40 Gy	9.7		16.5	
	< 59.4 Gy	8.9	0.833	16.5	0.837
	> 59.4 Gy	11.5		18.8	
Bilateral dose	< 40.8 Gy (median)	9	0.72	16.6	0.961
	> 40.8 Gy (median)	8.7		16.5	
	< 40 Gy	8.8	0.277	16.4	0.645
	> 40 Gy	9		16.9	
	< 59.4 Gy	8.9	0.862	16.5	0.312
	> 59.4 Gy	12.5		16.9	
SVZ dosimetric data from the GTR group					
Ipsilateral dose	< 46.4 Gy (median)	9.4	0.707	17.7	0.590
	> 46.4 Gy (median)	9.8		17	
	< 40 Gy	9.5	0.770	19.1	0.576
	> 40 Gy	9.7		17.1	
	< 59.4 Gy	9.5	0.225	17.2	0.567
	> 59.4 Gy	10.1		18.1	
Contralateral dose	< 23.4 Gy (median)	10.4	0.735	17.6	0.748
	> 23.4 Gy (median)	9.4		17	
	< 40 Gy	9.5	0.552	17.6	0.579
	> 40 Gy	9.7		17	
	< 59.4 Gy		-		-
	> 59.4 Gy				
Bilateral dose	< 34.9 Gy (median)	9.7	0.826	17.2	0.609
	> 34.9 Gy (median)	9.1		17.8	
	< 40 Gy	9.3	0.222	17.2	0.823
	> 40 Gy	10.8		17.5	
	< 59.4 Gy		-		-
	> 59.4 Gy				

For the multivariate analysis, stability of the model required the creation of different CPH models for each SVZ volume. Patients with multifocal tumors had significantly worse PFS, and patients with low KPS and minimal resection had significantly shorter OS (Table 4).

**Table 4:** Multivariate analysis. * Entered as a continuous variable. SVZ: Subventricular zone; STR: Subtotal resection; GTR: Gross total resection.

	Progression-free Survival				Overall Survival		
	HR	95% CI	P-value		HR	95% CI	P-value
Ipsilateral SVZ							
Age (> 50 years vs. < 50 years)	1.24	0.95–1.63	0.11		1.10	0.84–1.43	0.50
Karnofsky score (< 70 vs. > 70)	1.29	0.98–1.69	0.07		1.51	1.16–1.96	<0.01
Resection (STR vs. GTR)	1.05	0.81–1.36	0.70		1.03	0.80–1.32	0.83
Resection (biopsy vs. GTR)	1.19	0.83–1.71	0.33		1.62	1.15–2.28	<0.01
Multifocality (yes vs. no)	1.51	1.11–2.04	<0.01		1.31	0.98–1.76	0.08
Total dose (> 60 Gy vs. < 60 Gy)	0.85	0.59–1.22	0.37		0.85	0.59–1.22	0.37
Ipsilateral SVZ dose*	1.00	0.99–1.01	0.70		1.00	0.99–1.01	0.69

## Discussion

This study found that for patients treated with long-course CRT, dose to the SVZ was not associated with PFS or OS. Furthermore, no benefit to increasing SVZ dose was observed when analysis was restricted to patients with GTR, a subgroup identified by a previous study as uniquely benefitting from high SVZ dose [26].

Our results are contrary to prior studies that suggested higher dose to the SVZ improves outcome [20, 22-26]. However, these studies were small, and used variable inclusion criteria. Some failed to address known prognostic factors such as performance status [25] and adjuvant chemotherapy [25, 26]. Exclusion of multifocal tumors [25, 26] is also questionable, since multifocality is characteristic of stem-cell-derived tumors [14, 16] and often confers poor prognosis [29]. It is worth noting that our findings are applicable to patients receiving combined radiation and temozolomide; thus, conclusions about the effectiveness of SVZ irradiation without concurrent chemotherapy cannot be made. Our study population is similar to others in terms of the incidental dose received by the SVZ. The mean dose received by the ipsilateral SVZ in our cohort was 47.8 Gy, which is very similar to Chen et al., at 49.2 Gy, and Lee et al., at 48.7 Gy. Due to the size of our study, our analysis had a larger group, n = 32, receiving a dose greater than 59.4 Gy. This is important because it allows for a more robust comparison with those receiving a lower dose. Indeed, Lee et al., which is the second largest study looking at this issue, had only 21 individuals in their high dose group.

Discrepancy exists among studies of SVZ irradiation. Although some demonstrate benefit [20, 22-26], others have found no association between dose to the SVZ and outcome [18], and one conversely suggested high SVZ dose was associated with worse prognosis [17]. There is also disagreement within the positive studies as to which SVZ target volume may be associated with outcome (ipsilateral [22, 23, 25, 26], contralateral [22], or bilateral [24]).

The strengths of our study include its large size, with patients being treated at a single institution using a similar treatment protocol. Our study is limited by the retrospective nature of our data. We also employed very strict selection criteria, which has the potential to introduce selection bias. However, this was done to ensure our subjects would be comparable to those in prior studies of SVZ. Also, strict selection criteria were necessary to prevent further sources of bias. For example, subjects who did not complete radiotherapy were excluded; this was necessary, otherwise patients who deteriorated significantly during treatment would have inherently received a low SVZ dose, conceivably introducing further bias to the data. Chemotherapy compliance is potentially influenced by a number of factors (patient preference, toxicity, early tumor progression, etc.) which cannot be controlled in the retrospective setting, and although another important prognostic factor, it was not possible for this to be assessed. The most significant limitation of the present study is the lack of molecular marker data. It is well established that certain molecular features, such as IDH1 and MGMT status, can be prognostic and/or predictive of outcome [19]. Unfortunately, during the time period when study population was treated the institution did not routinely obtain molecular marker data on patients with GBM. It is certainly possible that SVZ dose could potentially have differential effects in the setting of different molecular phenotypes.

Targeting stem cell reservoirs may not be clinically beneficial, and can lead to larger volumes of brain tissue receiving radiation dose. This is important because current phase II trials are deliberately including the SVZ in their target volume [30]. The SVZ is anatomically close to the hippocampal formation, which is a structure important for memory and cognition that is being purposely avoided in studies using modern radiotherapy techniques [27, 28]. In the future, it would be worthwhile for similar studies to include tumor genetics in their analysis.

## Conclusions

The results of this study suggest that prognosis is unrelated to dose received by the SVZ. Indeed, prospective data are required to determine the value of SVZ irradiation in the treatment of GBM. However, trials deliberately including this region as a target volume should be done with caution.
